# Influence of Equal Channel Angular Pressing and Cyclic Extrusion Compression on the Microstructure Evolution and Mechanical Properties of Pure Aluminum

**DOI:** 10.3390/ma17205061

**Published:** 2024-10-17

**Authors:** Mohamed Ibrahim Abd El Aal

**Affiliations:** Mechanical Engineering Department, College of Engineering at Wadi Addawaser, Prince Sattam bin Abdulaziz University, Wadi Addawaser 18734, Saudi Arabia; mi.abdelaal@psau.edu.sa

**Keywords:** equal channel angular pressing (ECAP), Cyclic extrusion compression (CEC), load–displacement, microstructure, tensile properties

## Abstract

The influence of equal channel angular pressing (ECAP) and cyclic extrusion compression (CEC) of Al-1080 on the pressing load, microstructure, and tensile properties was investigated. The pressing peak loads of the CEC were 218.8–265.4% higher than those of the ECAP, with a more complex load behavior in the CEC process. The deformation morphology of the ECAP samples indicates an improvement in the deformation homogeneity with the number of passes. Shear band morphology with a decrease in the shear band width from the center to the outer surface makes up the predominant pattern of the CEC samples. The ECAP samples have 13.9–44% smaller average grain sizes, with 3.8–8.1% higher high-angle grain boundaries (HAGBs) than the CECed samples. The ECAP and CEC processing improve the tensile strength. However, the ECAP sample’s tensile strength (UTS) and the proof strength (σ_0.2_) were 11.5–20.6 and 2.6–16.4% higher than that of CEC, without noticeable differences in elongation. The σ_0.2_ values were predicted accurately with a deviation range of 1.8–7.3% from the experimental one. The ECAP samples are easy to process under lower loads. Moreover, ECAP samples have an equiaxed grain microstructure with a higher degree of deformation homogeneity and tensile strength.

## 1. Introduction

Producing cylindrical mechanical parts such as shafts and bars that are both lightweight and high-strength is an aim of different applications to reduce power consumption. The fabrication of such mechanical parts with ultrafine (UFG) or nanograined structured materials and high strength has been a primary goal over the past 20 years. The production of UFG or nanograined materials was achieved using the severe plastic deformation (SPD) technique. The SPD technique includes many processes, such as ECAP and high-pressure torsion (HPT) [1,2,3].

ECAP is thought to be the most efficient SPD procedure to produce large bulk samples with a circular cross-section that may be used in various industrial applications [4]. The details of the principles, the routes, and the calculations of the strain imposed in the ECAP were investigated [5,6,7]. The importance of Al and its alloys makes it the primary concern of ECAP processing of different pure and alloy samples of Al [8,9,10,11,12,13,14,15,16].

Cyclic extrusion compression (CEC) is considered the most probable process for producing large cylindrical samples, similar to the ECAP process. Interestingly, both the ECAP and CEC processes can save the initial dimension of the cross-section of the processed samples. The ECAP and CEC processes are performed through pressing by special dies that impose large strains that contribute to grain refinement. However, ECAP is performed under shear strain. In the case of the CEC, the samples are deformed through the extrusion process combined with compression, which conserves the cross-section of the sample.

The great efforts made by M. Richert resulted in achieving a new SPD process through deformation by repetitive extrusion under compression [17]. The details of the CEC, parameters, and die construction can be viewed in previous works [17,18,19,20]. The CEC of Al and its alloys are the focus of most previous works [17,18,19,20,21,22,23,24]. The evolution of the microstructure and the saturation in grain size that affects the microhardness and yield strength were also investigated [18,19,20,21,22]. The saturation phenomena can be defined as the stability of the hardness and compressive yield strength values of the CECed sample at an accumulated strain with no further increase in their values, even after processing under a higher strain. The hardness and compressive yield strength saturation values depend on the imposed strain per cycle and the processed material [18,19,20,21,22].

However, the saturation phenomena during CEC cannot be generalized [23,25,26,27,28]. The CECed AlCu4Zr, AlMg5, Al-6082, Al-Si-Cu, Al-6061, and AA5052 alloys grain sizes continue to decrease, which causes the hardness and strength to increase with further processing [23,25,26,27,28]. The hardness of AlCu4Zr and AlMg5 alloys initially increased by 49 and 26% after the first cycle, then it increased by 3–7% and 3–35% [23]. The Al-6082 and Al-Si-Cu grain sizes were decreased by 20–38% and 7–30% with increasing cycles [25,26]. Moreover, the hardness of CECed Al-6061 increased by 45% after one cycle, then by 12–8% with further processing [27]. Similar behavior was observed in the increase in hardness of Al-6061-5 and 10% SiC with the same number of cycles [27]. Furthermore, the ultimate and yield tensile strengths of the AA5052 alloy increase by 46 and 24% after the first cycle and then by 26–39% and 12–6% after processing up to 2–4 cycles [28]. Therefore, the saturation of the mechanical properties cannot be taken for granted during the CEC.

The previous works of ECAP and CEC of Al and its alloys were performed individually. However, no detailed comparison between their influence on the microstructure and mechanical properties has been found until now. Therefore, new studies are still needed to select the most appropriate SPD method to produce nano or UFG-structured cylindrical mechanical parts. There is an obvious need for new work on Al processing using ECAP and CEC, or a comparison between them, due to the shortage of recent works on this subject. A few previous works published after 2020 can be found about the ECAP and CEC processing of Al. Interestingly, the recent works concentrated mainly on magnesium, which cannot be used in comparison with Al.

In addition to the shortage of comparisons between the effect of the ECAP and CEC processing of Al samples in previous works, a new study is also needed to trace the effect of the processes on the applied load behavior, the deformation morphology, and the mechanical properties under different numbers of passes and cycles. Furthermore, there is a need to use a mathematical model in calculating the tensile strength that reduces the number of samples needed, and, therefore, a need for experimental tests that consequently reduce the overall cost of the research. Therefore, a new study providing a brief comparison between the ECAP and CEC processing of Al samples is required to find the most effective process in producing cylindrical parts with UFG and nano microstructures.

The current study goals are as follows. First, we search for the ease in application and repetition of the ECAP and CEC processes. Second, we study the effect of ECAP and CEC under the same imposed strain per each pass or cycle on load–displacement behavior and peak load required. Third, we investigate the tracing of the microstructure evolution. Fourth, we study the influence of ECAP and CEC on the tensile properties and predict the values of the tensile strength mathematically. Fifth, we select the most appropriate SPD process for producing nano- or UFG-structured Al cylindrical parts.

## 2. Materials and Methods

**Figure 1:**
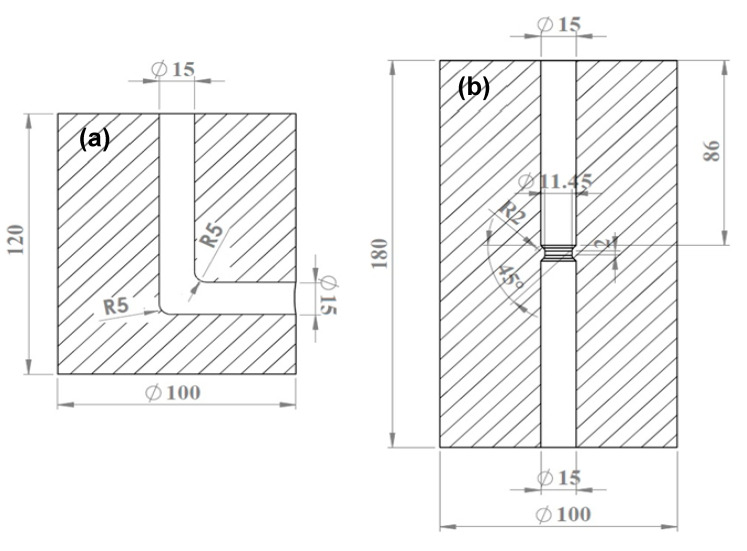
Drawing of ECAP and CEC dies (unit: mm). (**a**) ECAP; (**b**) CEC.

Al-1080 bars with 15 mm diameter and 80 mm length, with the chemical composition indicated in Table 1, are used. A split ECAP die with a channel of 15 mm diameter, corner radius (R) of 5 mm, inner (Φ) of 90, and outer (Ψ) die angles of 15°, shown in Figure 1a, is used. The ECAP die imposed a strain of 1.08 per pass according to the IWAHASHI Y Equation (1), using route A [5,6,7].

(1) $$\varepsilon_{\mathrm{ECAP}\;}=N\left[\frac{2cot\left(\frac{\Phi }{2}+\frac{\Psi }{2}\right)+\Psi cosec\left(\frac{\Phi }{2}+\frac{\Psi }{2}\right)}{\sqrt{3}}\right]$$

where Φ, Ψ, and N are the inner and outer die angles and number of passes. ECAP is performed up to 10 passes at room temperature (RT) under the speed of 1 mm/s using zinc soap as a lubricant [5,7,12,15,16,29,30] by double-actuating hydraulic press with a maximum pressing capacity of 200 tons.

**Figure 2:**
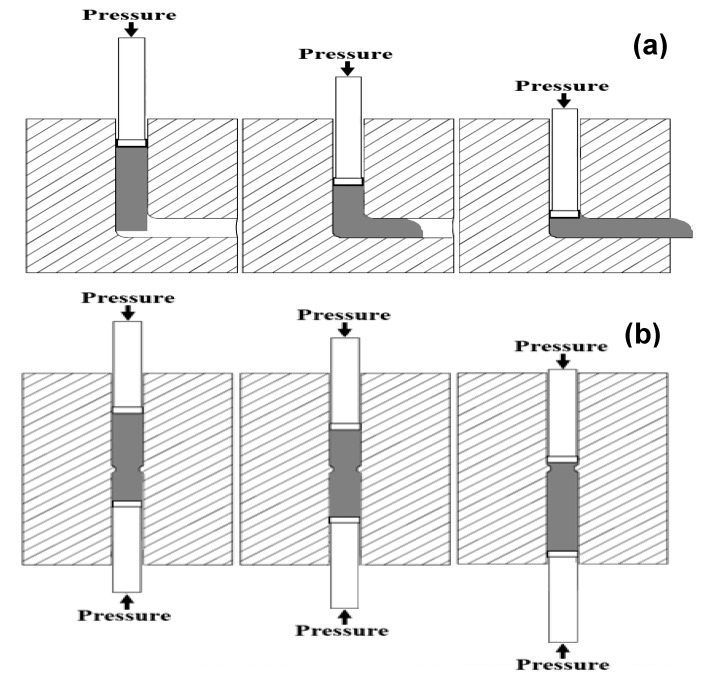
Schematic diagram of the deformation of the Al 1080 samples of the (**a**) ECAP and (**b**) CEC processing at the start, during, and the end of each process.

**Figure 3:**
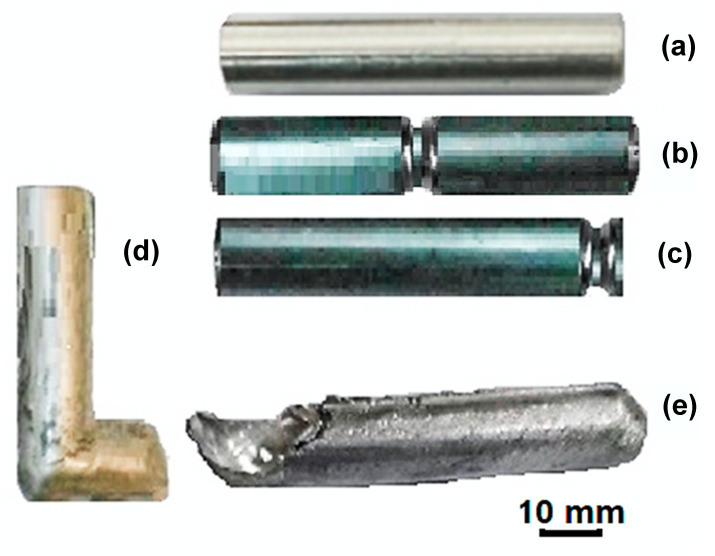
Macrographs of (**a**) Al-1080 machined sample, (**b**) CEC sample during the deformation process, (**c**) CEC sample final shape, (**d**) ECAP sample during the deformation process, and (**e**) ECAP sample final shape.

A solid die performed the CEC, as shown in Figure 1b. Al-1080 bars with diameters and lengths of 15 and 80 mm were CECed. The bars were extruded from 15 to 11.45 mm, then their diameter was restored to 15 mm under applied pressure from the lower punch. The CEC die can give strain on the sample = 1.08 per CEC cycle, according to Equation (2) [17,18,19,20].

(2) $$\varepsilon_{\mathrm{CEC}\;\;\;}=4n\ln\left(\frac{d_{0}}{d_{m}}\right)\;$$

where d_0_ and d_m_ are the diameters before the CEC process and during the extrusion step. In the current study, d_0_ and d_m_ are equal to 15 and 11.45 mm. The CEC die with a die angle of 45° and a corner radius (R) of 2 mm was used to achieve a high degree of deformation homogeneity [31]. The CEC process was carried out for up to 10 cycles with a total imposed strain of 10.8 under the same conditions as the ECAP using the same hydraulic press. Figure 2 shows the deformation behavior and steps of the Al-1080 samples during the ECAP and CEC. Moreover, macrographs of the Al-1080 samples before and after the ECAP and the CEC are shown in Figure 3. The load–displacement during the ECAP and CEC processing was recorded to obtain their curves and the peak load for each process.

The deformation morphology was traced using an optical microscope (OM) parallel to the longitudinal direction (pressing direction) through a length of 15 mm from the middle of the samples. The samples were ground with SiC emery papers from 100 to 2000 successively and then polished using Al_2_O_3_ of 0.05 µm. The samples were then cleaned with a distilled water bath, followed by cleaning in an ethanol bath using an ultrasonic apparatus. Finally, the samples were dried and etched using Keller’s etchant for 10–30 s.

The Al-1080 samples before the SPD processing were sectioned in both the longitudinal and transverse directions and prepared in the same way used in the case of the deformed samples to measure the initial grain size. An OM model NIKON with a computerized camera was used for the examination. The measurement of the shear bands width and initial grain size was carried out using the line intercept method using the software Lince242udt (version 2) particle size.

Scanning electron microscopy associated with the electron backscatter diffraction technique (SEM/EBSD) is used to measure grain size and grain boundary misorientation angles and to produce grain boundary maps. A 3-dimensional Total Analysis System (Dual Beam FIB) equipped with EBSD (Link EDAX system) and associated software (OIM 4.5) was used. The step size of 20 nm was used in the mapping process. All points with a confidence index (CI) lower than 0.1 were cleaned to use more reliable and correct data. Furthermore, the misorientation angles of less than 2° were excluded from the EBSD data analysis. The grain sizes under five times the step size were excluded from the grain size calculations. The OM samples were further polished using diamond paste sprays of 0.25 µm and colloidal silica and used as EBSD samples.

X-ray diffraction (XRD) measurements of the samples were performed using a D/max-r B X-ray diffractometer with Cu Kα radiation and the wavelength of 1.5418 A over a 2θ range of 10–100° to determine the average dislocation density ρ. The ρ was calculated using the Rietveld method previously mentioned [16,32,33]. Tensile samples with 5 mm diameter and 20 mm gauge length, according to (ASTM E8), were machined parallel to the longitudinal direction of the samples. The tensile test was conducted up to failure at RT and strain rate of 8.33 × 10^−3^ s^−1^ using a TESTO METRIC 200 kN machine. The tensile was repeated five times for each condition. 

## 3. Results and Discussion

### 3.1. The Ease in Performance and Repetition of the ECAP and CEC Processes

The ECAP process was easier and faster to repeat than the CEC. Therefore, the ECAP processing up to multiple passes and its die cleaning and lubrication between passes can be performed quickly and easily. The CEC processing up to multiple cycles was more complex and consumed a long time. Adhesion between the CEC samples and the die was noted, even with lubrication. Therefore, unscrewing and reassembling the CEC die required a longer time than that of the ECAP one. The ECAP and CEC samples have some unusable parts, such as the tail and head for the ECAP sample and the neck in the CEC one. However, both samples have approximately the same length after removing the unusable parts, as shown in Figure 2.

### 3.2. The Load−Displacement Curves

**Figure 4:**
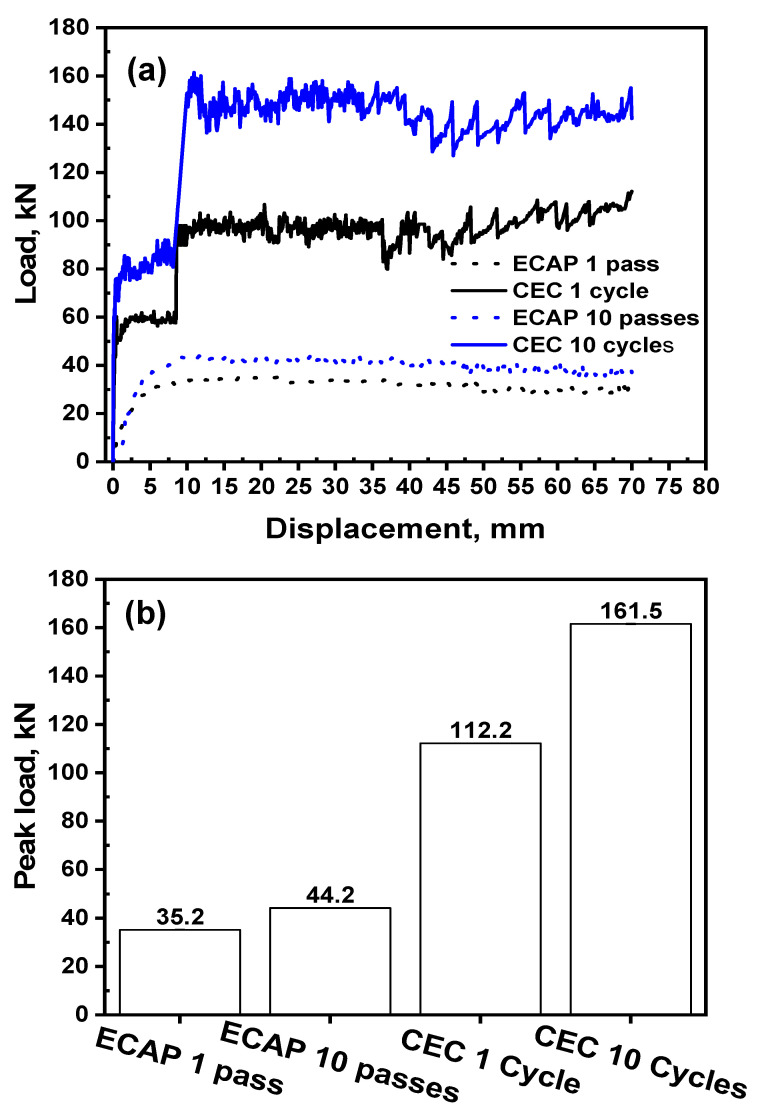
(**a**) Load–displacement curves and (**b**) peak load of Al-1080 samples processed by ECAP and CEC.

Figure 4a shows the load–displacement curves of the ECAPed and CECed Al-1080 samples. The ECAP load increases as the sample passes through the shear zone, and then the curve is characterized by a high peak load, a ramp, and a steady state area with a decrease in the load. The ECAP load curve behavior is similar to that previously noted of ECAPed Al-1080 [34]. The ECAP load curve behavior is due to the effect of Ψ angle value. The occurrence of a high peak load with ramp load–displacement curves depends mainly on the ECAP die with the Ψ < 40° [35,36]. Therefore, in the current work with an ECAP die with geometry with a Ψ value of 15°, which is <40°, the load curve is characterized by the observed shape.

The ECAP load–displacement curve behavior after ten passes was similar to that after one pass with a higher peak load. The peak load of the ECAP was increased from 35.2 kN after one pass to 44.2 kN with a percent increase of 25.6% after ten passes, as shown in Figure 4b. The peak loads of the ECAPed Al-1100, Al-3004, and Ti samples increased by 30, 56, and 25.7% after six, eight, and four passes [14,37], which is close to the current results. The increase in ECAP peak load is due to the increase in deformation resistance after each pass [37]. Moreover, the imposed work hardening properties of the sample and strength increase due to ECAP processing, as previously noted [14,37,38].

The CEC load–displacement curve differed from the ECAP’s, as shown in Figure 4a. The CEC load increased as the extrusion process started up to 52.5 kN, and then the load continued in a steady state area (for the distance approximately equal to the extrusion neck). With further pressing, the samples exited the extrusion and started to be compressed by the lower punch in the compression step, contributing to a sudden increase in the load to 112.2 kN. The load was then oscillated around the peak load value and continued in oscillation until pressing was completed. Interestingly, the extrusion step load during the CEC with an extrusion ratio of 1.72 and load of 58.5 kN was close to that noted during the direct extrusion of Al-1080 using an extrusion ratio of 2 of 55 kN [34].

The CEC load curve still had the same behavior after ten cycles. The extrusion and compression loads increased to 83.7 and 161.5 kN after CEC for 10 cycles, as shown in Figure 4a. The CEC up to ten cycles increased the extrusion and peak loads by 43.1–43.9%. This increase is due to the rise of the deformation resistance after each cycle, imposed work hardening properties, and the strength increase. The oscillation of the load was more obvious in the CEC process than in the ECAP due to the complexity of the CEC and higher friction during the extrusion step combined with compression. 

Interestingly, the load oscillation range has approximately the same value under the different CEC number of cycles with a range of 30 kN. The CEC load–displacement curve behavior in the current study under various numbers of cycles was similar to those obtained through finite element simulations (FEM) and experimental work [39,40,41]. However, the extrusion and compression steps load ratio of 92% was lower than that of 500–400, 350–360, and 178–160% of CECed Mg alloys noted through FEM and experimentally [39,40,41]. This difference is due to the material’s properties and CEC die geometry [39,40,41].

The comparison between the ECAP and the CEC indicates the following. The ECAP load curves were smoother than those of the CEC process under the same imposed strain. The ECAP die shape with the processing pass contains only the shear process at the shear zone. The ECAP load curves are characterized by an increase in load, a ramp, and a steady state area with a load decrease. Thus, ECAP curves were free from sudden changes in the load values.

The CEC load curves are characterized by different steps with an increase in load due to the extrusion step. Then, a steady state area is approximately equal to the neck of the extrusion step, followed by a sudden increase as the compression occurs by the lower punch that continues until the process completion. Therefore, the load path in the CEC process is considered more complex than the ECAP. The complexity of the load pass in the CEC process is due to the change in the sample cross-section area in the extrusion step. The extrusion step loads in the CEC process of 58.5 and 83.7 kN after 1 and 10 cycles were higher by 66.2 and 89.4% than the peak load of the ECAP after 1 and 10 passes. This observation was also noted by comparing the extrusion and ECAP processing loads [34]. The sudden increase in the load due to the compression step also increases the curve complexity. Interestingly, the peak loads of the CEC of 112.2 and 161.5 kN after 1 and 10 cycles shown in Figure 4b are higher by 218.8 and 265.4% than those of the ECAP up to 1 and 10 passes. Therefore, ECAP processing can be more effective than CEC due to the lower required load and smoother load–displacement curve that saves the tool (dies and punches) from fast failure.

### 3.3. Microstructure Analyses

#### 3.3.1. Optical Microscopic Observations of the Deformation Morphonology (Shear Bands)

**Figure 5:**
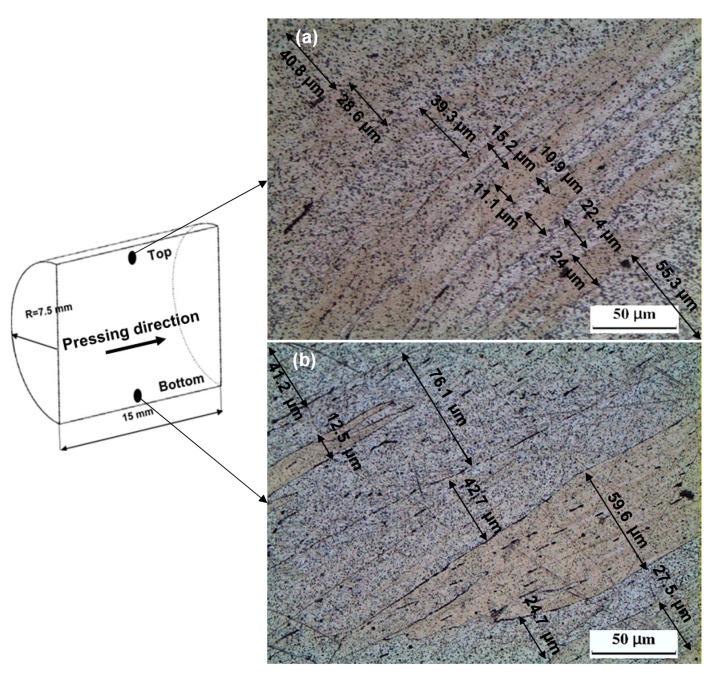
OM micrographs of the ECAP processed sample up to 1 pass in longitudinal direction (pressing direction) at (**a**) top and (**b**) bottom.

**Figure 6:**
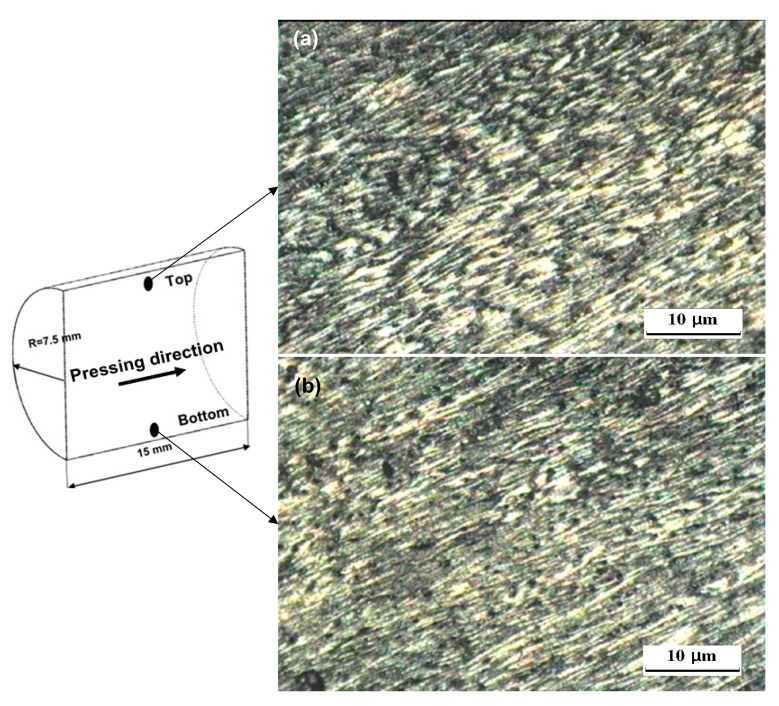
OM micrographs of the ECAP processed sample up to 10 passes in longitudinal direction (pressing direction) at (**a**) top and (**b**) bottom.

**Figure 7:**
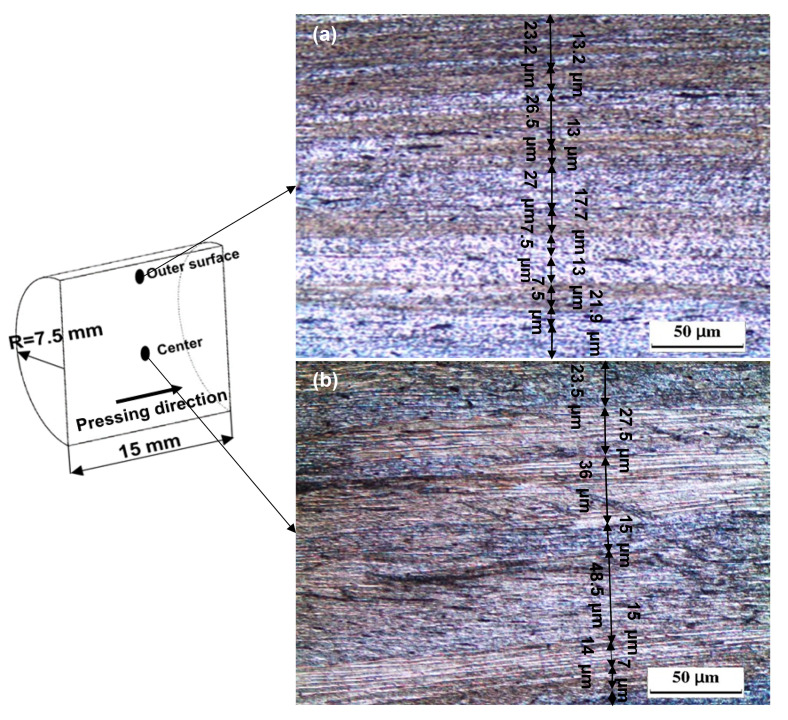
OM micrographs of the CEC processed sample up to 1 cycle in longitudinal direction (pressing direction) at (**a**) outer surface and (**b**) center.

**Figure 8:**
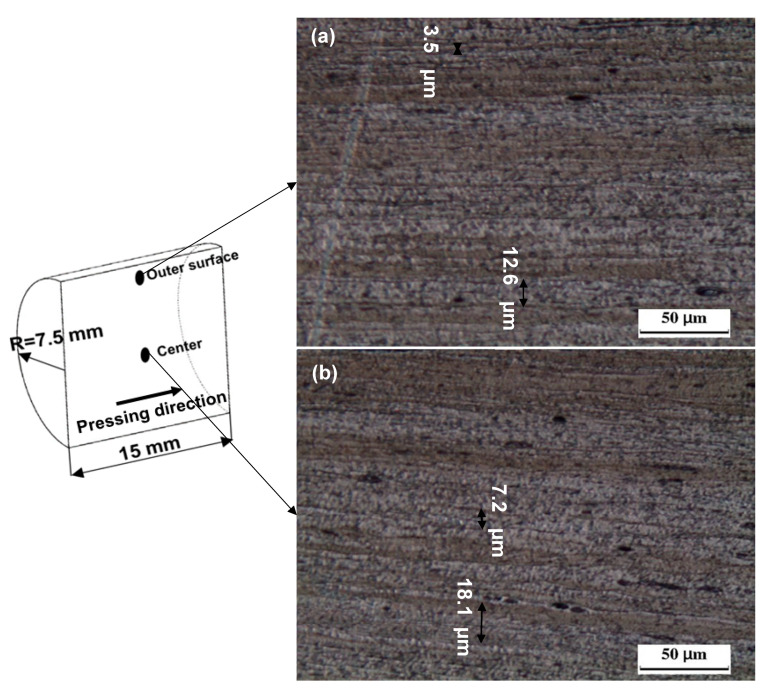
OM micrographs of the CEC processed sample up to 10 cycles in longitudinal direction (pressing direction) at (**a**) outer surface and (**b**) center.

The OM observations of the ECAP and CEC samples in the pressing direction are shown in Figure 5, Figure 6, Figure 7 and Figure 8. The sample is covered with shear bands parallel to the shear plane after ECAP for one pass, as shown in Figure 5. The shear bands width at the bottom of the samples varied from 76.1–12.5 μm, as shown in Figure 5a. Moreover, the shear bands width at the top, as shown in Figure 5b, varied from 55.3–10.9 μm. Therefore, the shear bands width at the top of the ECAP sample was smaller than that at the bottom, as previously noted during the ECAP of Al-1080 and Cu [34,42,43]. The difference in the shear bands width can be explained by the corner gap formation at the bottom of the sample [12,16,34,44,45]. The ECAP sample morphology was changed, and the width of shear bands decreased, or, even, the shear band disappeared across the sample after ten passes, as shown in Figure 6. Therefore, the ECAP further processing improves the deformation homogeneity [10,12,15].

The ECAP and CEC samples processed up to one pass and cycle had similar features, as shown in Figure 5 and Figure 7. However, the shear bands in the case of the CEC samples were parallel to the extrusion direction. The shear bands width after one cycle increased from the outer surface to the center, as shown in Figure 7a,b. The shear bands width at the outer surface after one cycle varied from 27 to 7.2 μm with an average of 16.1 μm, as shown in Figure 7a. The range of the shear bands width and their average width were increased to 48.5–7 μm and 23.3 μm at the center, as shown in Figure 7b. The smaller shear bands width at the outer surface of the CEC sample after one cycle is due to the high friction at the outer surface [34]. Moreover, the decrease in the imposed strain values from the center to the outer surface of the extruded and CEC samples produces thinner shear bands at the outer surface [31,34,39,40].

The CECed Al samples [18,19,20,21,22] have the same morphology noted in the present work. Unfortunately, the variation in the shear bands width from the center to the surface of the CEC samples has not been mentioned [18,19,20,21,22]. However, the extrusion can be considered the closest process to the CEC. The decrease in the shear bands width from the center to the surface was also noted in the extruded Al-1080 and Al-6061 samples [34,46]. The CEC of the Al-1080 under an extrusion ratio of 1.72 with an imposed strain of 0.54 combined with compression increases the total strain per CEC cycle to 1.08. The average shear bands width after CEC for one cycle was 16.1 and 23.3 μm at the outer surface, and the center was smaller than those of 69.6 and 52 μm of extruded Al-1080 under an imposed strain of 0.7 [34]. Therefore, the CEC process can impose a higher strain than the traditional extrusion while conserving the initial sample size.

The shear bands structure was still observed after the deformation under strain of 10.8, as previously observed [18,20], and is shown in Figure 8. Shear bands were heavily deformed, with a decrease in shear bands width to 12.6–3.5 μm, as indicated by arrows in Figure 8a, with an average of 8.1 μm at the outer surface after ten cycles. Moreover, the shear bands width with a range of 18.1–7.2 μm, as indicated by arrows in Figure 8b, with an average width of 11.9 μm, was observed at the sample center. The decrease in the shear bands width with the number of CEC cycles was previously noted [18,20,22]. Although both the ECAP and CEC contribute to the shear bands width decrease with further processing, the ECAP processing contributes to the disappearance of the shear bands. 

#### 3.3.2. Microstructure Evolution

**Figure 9:**
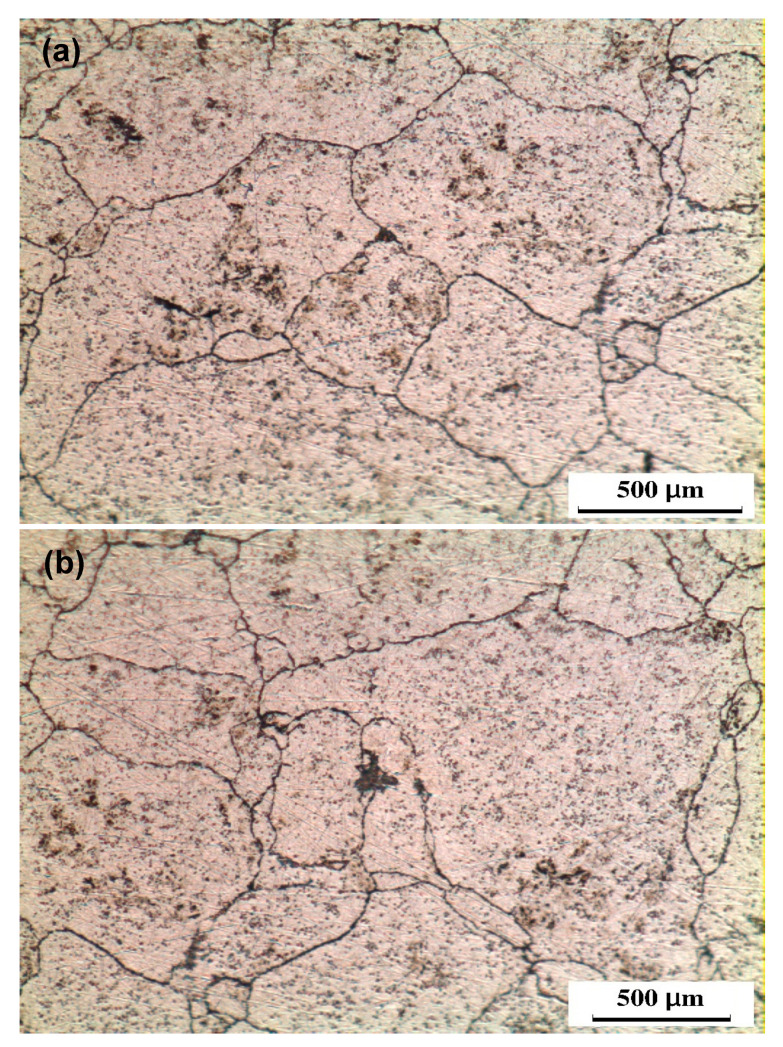
OM micrographs of the Al-1080 sample in the (**a**) longitudinal and (**b**) transverse directions.

**Figure 10:**
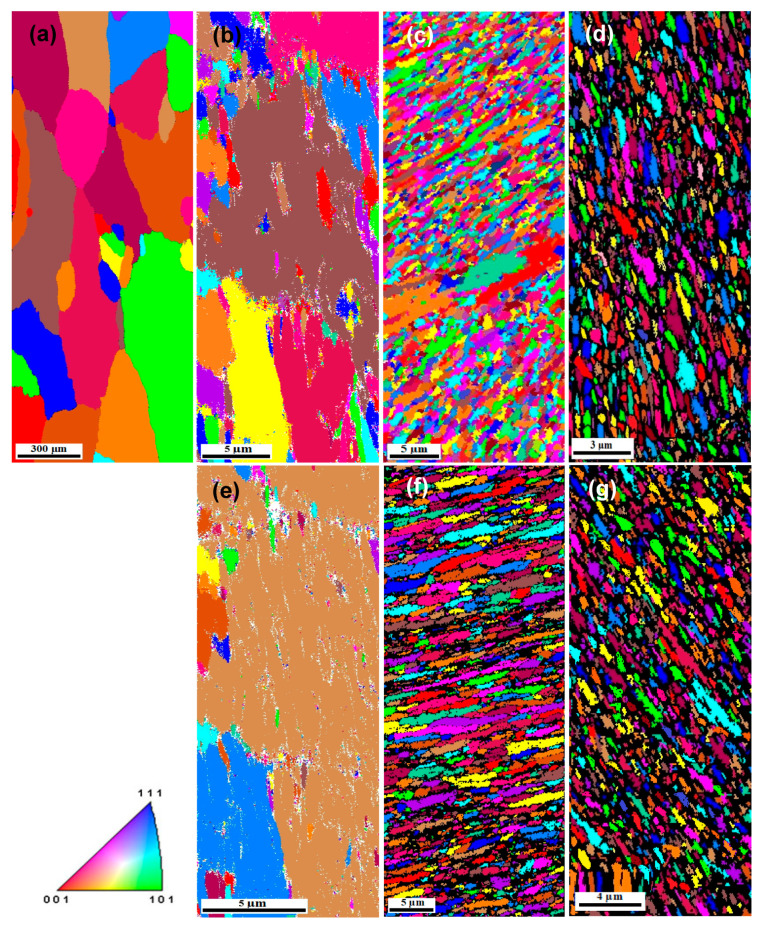
Color-coded orientation map images of (**a**) Al-1080 sample [16], (**b**–**d**) samples processed by ECAP up to 1, 5, and 10 passes, and (**e**–**g**) samples processed by CEC up to 1, 5, and 10 cycles.

Figure 9 shows the OM observations of the Al-1080 samples in the longitudinal and transverse directions. The microstructure of the Al-1080 consists of equiaxed grain in both directions with average grain sizes of 415.2 and 414.9 µm. Interestingly, the Al-1080 samples microstructure obtained using EBSD in the longitudinal direction have an approximately equiaxed grain, with an average grain size of 414 µm, as shown in Figure 10a, which confirms the OM results. The microstructure was transformed into elongated grains after one pass, as shown in Figure 10b and noted previously [9,10,16]. The average grain size after ECAP for one pass was 19.8 µm, with a grain size range of 1.39–22.66 µm, as indicated in Table 2. The obtained average grain size was close to that of ECAPed Al-1080 for one pass of 20 µm [16].

The microstructure of the ECAP sample evolved into a mixture of elongated and equiaxed grains after five passes, as shown in Figure 10c and previously noted for Al-1050 and 1060 after four passes [9,10]. The average grain size and range decreases to 1 µm and 0.42–4.49 µm after five passes, as shown in Table 2. After ten passes, the grains were changed into an approximately equiaxed grain structure, with an average of 0.3 µm and a size range of 0.15–0.84 µm, as shown in Figure 10d and Table 2. The grain refinement occurred due to the shear strain imposed during the ECAP [1,2,5,6,7]. The shear strain imposed along the intersection of the ECAP die to channels contributes to the grain refinement that continues with an increase in the number of passes. Nonindexed areas (black areas) were noted, with an increase in the passes up to 10. Therefore, the high imposed strain leads to further grain refinement, so grain sizes lower than 100 nm are present. Microstructures with equiaxed grain structures were obtained in the case of ECAPed Al-1050, 1060, and 1080 [9,10,12,15,29]. The ECAP of the Al-1050 decreases the grain size from 50 µm to 9.7, 9.2, and 0.85 µm after 2, 4, 8, and 16 passes [9]. Furthermore, the grain size was decreased to 1.65, 0.64, and 0.36 µm after the ECAP of Al-1060 to one, four, and eight passes [10].

An elongated grain structure with an average grain size and range of 23 and 1.8–27.9 µm was noted, as shown in Figure 10e and Table 2, after CEC for one cycle. The observed structure is the main pattern of the microstructure of CECed Al-1050, Al99.99, Al99.992, AlMg5, AlCu4Zr, and AlMgSi (Al-6082) noted previously [18,19,20,21,22,23,24,25]. Moreover, the OM observations of the CECed sample after one cycle, shown in Figure 7, agree with the obtained microstructure and that previously noted after the extrusion operation [34,46]. The microstructure features of the CEC sample after five cycles are the same as those after one cycle, with an average grain size and range of 1.8 and 0.53 to 4.86 µm, as shown in Figure 10f and Table 2. However, increasing the imposed strain to 5.4 contributes to the appearance of a percent of equiaxed grains, but smaller than observed in the ECAPed sample after five passes. The formation of the equiaxed grains is due to the intersection of shear microbands, dividing them into almost equiaxial subgrains and evolving into equiaxed grains, as previously observed [18,20,21,22]. The CEC to ten cycles decreases the average grain size and range to 0.38 µm and 0.21–0.96 µm, as shown in Figure 10g and Table 2. Increasing the number of cycles increases the possibility of the intersection of microshear bands, therefore, further dividing the microshear bands and the elongated grains into approximately equiaxial grains microstructure, as shown in Figure 10g and previously noted [18,20,21,22,23,25,26,27,28]]. Interestingly, nonindexed areas were also noted after CEC up to ten cycles, as in the case of the ECAPed samples after ten passes.

The previous works of the CEC of pure Al with different purity were not concerned with determining the grain size [18,19,20,21]. Considering the clear images of the microstructure of CECed Al99.99 obtained by transmission electron microscopy (TEM), only data about the spacing between boundaries were provided [22]. The interest of previous works focused on measuring the shear bands width. The microshear bands width of CECed AlMg5 was decreased from 0.27 µm to 0.21 µm, then to 0.19 µm, and, finally, to 0.156 µm under the strain of 2, 3, 8, and 14, respectively [23]. The microshear bands width of the CECed AlCuZr4 alloy also decreased from 0.22 µm to 0.135, and, finally, to 0.125 µm under the strain of 2, 6, and 14, respectively [23]. Recently, the CECed AlMgSi (Al-6082) and AlSi5Cu1 alloy’s grain size was determined effectively [25,26]. The CECed AlMgSi (Al-6082) grain size decreased from 0.223 to 0.176 µm, then to 0.12 µm, and, finally, to 0.07 µm under the strain of 2, 5, 10, and 16, respectively [25]. Furthermore, the CECed AlSi5Cu1 alloy grain size decreased from 0.235 µm to 0.21 µm 8, then to 0.179 µm, and, finally, to 0.127 µm under the strain of 1.8, 3.6, 8.9, and 13.4, respectively [26]. Equiaxial grains % increases with the ECAP and CEC number of passes and cycles. However, the ECAPed sample has a higher percentage of equiaxial grains, as shown in Figure 10.

**Figure 11:**
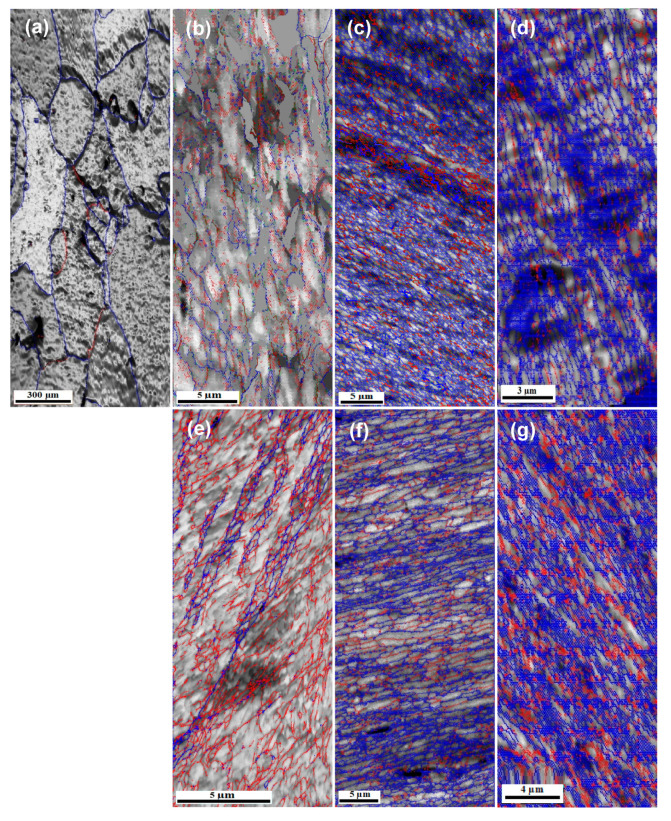
Color-coded grain boundaries map images of (**a**) Al-1080 sample, (**b**–**d**) samples processed by ECAP up to 1, 5, and 10 passes, and (**e**–**g**) samples processed by CEC up to 1, 5, and 10 cycles (high angle ≥ 15° blue color; low angle ≤ 15° red color).

The high-angle grain boundaries (HAGBs: misorientation angle ≥ 15°) and low-angle grain boundaries (LAGBs: misorientation angle < 15°) are highlighted in blue and red colors, as shown in Figure 11. The ECAP up to one pass indicates the presence of 40% of HAGBs and an average misorientation angle of 21.9°, as shown in Figure 11b and Table 2. The HAGBs grain boundaries percentage was increased to 75.8 and 83.2% after 5 and 10 passes, as noted in Table 2. Moreover, the average misorientation angle increases to 32° and 35.2° after 5 and 10 passes, as shown in Figure 11c,d and Table 2. Therefore, the increase in the ECAP number of passes contributes to evolving the microstructure into a UFG structure with HAGBs.

Similar observations of the evolution of the ECAPed pure Al microstructure into UFG with HAGBs were also noted previously [9,10,15]. The HAGBs percentage increased from 9% to 22%, then to 43% and 60% in the pressing direction of the ECAPed Al-1050 after 1, 4, 8, and 16 passes, respectively [9]. Interestingly, the increase in the HAGBs percentage was also noted in the plane normal to the pressing of the ECAPed Al-1050 [9]. The HAGBs percentage of ECAPed Al-1060 samples also increased from 8.4% to 39.2% and 79.3% with average misorientation angles of 7, 17, and 32.9° [10]. Therefore, the previous works [9,10,15] confirm the present results of evolving the pure Al microstructure into a UFG structure with HAGBs while increasing the ECAP number of passes.

Figure 11 shows the grain boundary maps of the CECed samples. The CECEed sample up to one cycle had 38.5% HAGBs with an average misorientation angle of 19.6°, as shown in Table 2. The CEC processing up to 5 and 10 cycles increased the percentage of the HAGBs to 73.3 and 77% with an average misorientation angle of 31° and 33.5°, as indicated in Figure 11f,g, and Table 2. Therefore, the CEC effectively produces UFG microstructure with HAGBs. The CECed Al 99.99%, AlMg5, AlCu4Zr, AlMgSi, and AA5052 microstructures evolved into UFG and nano microstructures with HAGBs [22,23,25,26]. The CEC of Al 99.99%, AlMgSi, and AA5052 under strain values of 0.9–6, 16, and 3.6 also produce a microstructure with HAGBs [22,25,26]. Moreover, the CECed AlMg5 and AlCu4Zr samples under strain of 13.86 have a microstructure with an average misorientation angle of 40° [23].

Although both ECAP and CEC processing can produce UFG microstructure Al-1080 samples with HAGBs, the ECAP under the same strain produces UFG samples with smaller equiaxed grains and a higher HAGBs %, which can be explained as follows. The EBDS maps shown in Figure 10 indicate the higher percent of the equiaxed grains in the ECAP samples relative to the CEC one, especially after 5 and 10 passes. Moreover, the ECAP to 1, 5, and 10 passes produces average grain sizes smaller by 13.9, 44, and 18.4% than that of the CECed samples up to 1, 5, and 10 cycles. Furthermore, Figure 11 and Table 2 indicate that the HAGBs % of the ECAPed sample up to 1, 5, and 10 passes was higher by 3.9, 3.8, and 8.1% than those of the CECed samples up to 1, 5, and 10 cycles, respectively. Moreover, the average misorientation angle of the ECAP samples was higher by 11.7, 3.2, and 5.1% than those of the CECed samples processed under the same strain. Therefore, ECAP is more effective in producing UFG Al samples with smaller grain sizes and higher average misorientation angles. 

### 3.4. Mechanical Properties

#### Tensile Properties

**Figure 12:**
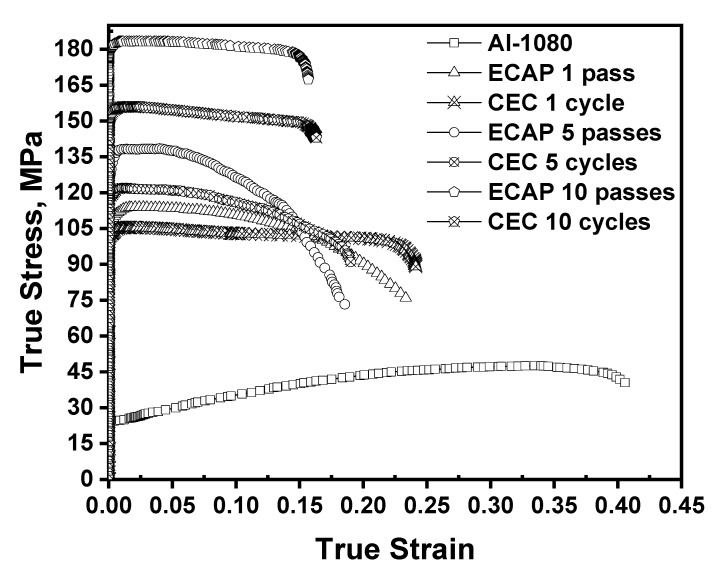
Tensile true stress–true strain curves of the different samples.

Figure 12 shows the tensile stress–strain curves of the different samples. The ultimate tensile strength (UTS) and the proof strength (σ_0.2_) were increased by 139.5 and 231.9% after ECAP for one pass. Then, the UTS and the σ_0.2_ increased by a lower rate of 21.5–32.4 and 27.4–32.1% after ECAP up to 5 and 10 passes. Therefore, the tensile strength was increased obviously after the first pass, and then at a lower rate with further ECAP processing.

The σ_0.2_ values of ECAPed Al-1080 and Al99.99 samples have the same behavior noted in the current work [12,13,15]. The tensile σ_0.2_ of Al-1080 and Al99.99 samples increased by 61.5 and 592.4% after ECAP’s first pass, then the rate of increase in the σ_0.2_ decreased to 1.9–13.7% and 1.5% after processing up to 6–10 passes using different routes [12,13,15]. The behavior of σ_0.2_ and yield strength of the ECAPed Al-1100 and 1070 [14,47] were close to that noted in the present work. However, the Al-1100 and 1070 samples indicate an increase in the σ_0.2_ and yield strength with percentages of 357.9 and 195.1% after one pass, then the rate of the enhancement of the σ_0.2_ and yield strength decreased to 3.1–14.1% and 11.6–8.8% after processing up to two and four passes. Finally, the σ_0.2_ and yield strength of the ECAPed Al-1100 and 1070 samples slightly decreased by 1.7 and 1.5% after the processing up to six and eight passes. Due to the very slight decrease in the σ_0.2_ and yield strength of the ECAPed Al-1100 and 1070 after the final pass, this can be considered negligible. Therefore, the previous works confirm the behavior of the σ_0.2_ in the present work with an increase in the number of passes [12,13,14,15,47]. Interestingly, the compression yield strength of the ECAPed Al-1050 and 1060 samples [8,9,10,11] have the same behavior of the tensile σ_0.2_ and yield strengths noted in the present and previous works [12,13,14,15,47]. The compression yield strength of the ECAPed Al-1050 and 1060 samples [8,9,10,11] increases obviously after the first pass, then with a lower rate with a further increase in the number of passes.

The UTS and σ_0.2_ of the CEC samples have approximately the same behavior as those of the ECAPed one. The UTS and σ_0.2_ of the CEC samples increased by 121.6 and 223.6% after the first cycle. With further processing, the UTS and σ_0.2_ of the CEC samples increased by 15.6–27.9% and 14.8–29.1% after 5 and 10 cycles. A similar behavior was noted after the CECed of AA5052, as the UTS and yield strength improved by 24 and 45.5% after the first cycle [28]. With further processing, the UTS and yield strengths of the CEC samples increased by 12–6 and 25.6–38.6% after the processing up to two and four cycles. The results of the CECed AA5052 [28] are the most relevant to the present work, as it depends on the tensile test.

The previous works of CEC of pure Al and its alloys built up their conclusion about the strength increase in the compression test results [20,21,22]. The compression σ_0.2_ of the CEC Al99.992 increased by 130.4, 11.3, and 3.4% after one, two, and four cycles [20]. However, the continuation of the straining did not contribute to a further increase in the σ_0.2_ value that saturated at the strain of four [20]. Interestingly, the decrease in the imposed strain per cycle during the CEC of the Al99.992 samples down to 0.2 per cycle decreased the rate of the increase in the compression σ_0.2_ to 75% after the first cycle [21]. Then, the compression σ_0.2_ increased by 157.2% after the two cycles and, finally, by 11.1 and 9.9% after 4 and 10 cycles, followed by a saturation at a strain of two [21]. The behavior of the saturation in the compression σ_0.2_ after a noticeable increase after the first cycle was also noted in the CECed Al-1050, Al-5Mg, and Al99.999 samples [21,22]. The saturation in the compression σ_0.2_ occurred at a strain of eight and five in the case of Al-1050 and Al99.999 [21,22]. On the other hand, two saturation steps observed for the CECed Al-5Mg occurred at strains 4 and 10 [21]. Therefore, the behavior of the σ_0.2_ of the ECAP and CEC samples can be considered close to each other under tensile load with no saturation.

The improvement in σ_0.2_ after the SPD processing is due to the grain refinement and grain boundary strengthening, as noted through the microstructure observations shown in Figure 10 and Figure 11. Moreover, further strengthening occurred through dislocation strengthening. The increase in the average dislocation density, after one pass or cycle from the value ρ < 0.1 × 10^14^ m^−2^ [48] to the values indicated in Table 3 (those are close to previously noted [16,47,48,49]), explains the high increase in the strength after one pass or cycle followed by an increase in a lower rate due to lower rate of the HAGBs and ρ increase. The σ_0.2_ was calculated through a mathematical model. The strengthening due to precipitation and friction stir welded was provided through different models by Starink et al. of different Al alloys [50,51].

A further modification by Qiao et al. for the previous models [50,51] produces a new one that can effectively calculate the σ_0.2_ of SPD Al samples [49] as indicated, in Equation (3). The Qiao et al. model was used in calculating the σ_0.2_ of SPD samples with high accuracy [16,49].

(3) $$\sigma_{0.2}=\sigma_{0}+\Delta \sigma_{\mathrm{gb}}+M\tau_{d}$$

where σ_0_ is the proof strength of samples (before deformation). The Δσ_gb_ is the strengthening by the size and boundaries of the grains and subgrain [51]. Moreover, M and $\tau_{d}$ are the Taylor factor and dislocation strengthening. The Δσ_gb_ can calculated as follows from Equation (4) [52,53].

(4) $$\Delta \sigma_{\mathrm{gb}}=\alpha_{2}\mathrm{Gb}\left[{\lambda f}_{\mathrm{sub}}\left(\frac{1}{\delta }\right)+\left(1-f_{\mathrm{sub}}\right)\left(\frac{1}{D}\right)\right]$$

where $\alpha_{2}$,  G,  b,  λ, ${\;f}_{\mathrm{sub}}$, δ, and D are constant, the shear modulus, Burger’s vector, the subgrain over grain boundaries strengthening contribution, the fraction of LAGB, subgrain size, and grain size, respectively. Finally, $\tau_{d}$ can be calculated as follows from Equation (5) [54].

(5) $$\tau_{d}=\alpha_{1}\mathrm{Gb}\sqrt{\rho }$$

where $\alpha_{1}$ is a constant. Thus, the general form of calculating $\sigma_{0.2}$ can be performed through the following Equation (6) [49].

(6) $$\sigma_{0.2}=\sigma_{0}+\alpha_{2}\mathrm{Gb}\left[{\lambda f}_{\mathrm{sub}}\left(\frac{1}{\delta }\right)+\left(1-f_{\mathrm{sub}}\right)\left(\frac{1}{D}\right)\right]+{M\alpha }_{1}\mathrm{Gb}\sqrt{\rho }$$

The values of all variables needed are listed in Table 3 based on the current and previous works [49,53,54,55,56].

**Table 3:** The strength model parameters.

Parameter	ECAP 1 Pass	ECAP 5 Passes	ECAP 10 Passes	CEC 1 Cycle	CEC 5 Cycles	CEC 10 Cycles	Reference of the Value
$\sigma_{0}\;$ **(MPa)**	31.5	31.5	31.5	31.5	31.5	31.5	Present work
$\;\alpha_{2}$	2	2	2	2	2	2	[53]
**G (GPa)**	26	26	26	26	26	26	[55]
**b (nm)**	0.286	0.286	0.286	0.286	0.286	0.286	[56]
**λ**	0.5	0.5	0.5	0.5	0.5	0.5	[16,49]
$f_{sub}$	0.6	0.242	0.168	0.615	0.267	0.23	Present work
δ **(μm)**	1.0	0.2	0.11	1.6	0.5	0.19	Present work
D **(μm)**	19.8	1.0	0.3	23	1.8	0.38	Present work
**M**	2.6	2.6	2.6	2.6	2.6	2.6	[56]
ρ **(×10^14^ m^−2^)**	1.22	1.5	1.8	1.2	1.5	1.8	Present work
$\alpha_{1}$	0.3	0.3	0.3	0.3	0.3	0.3	[54]

Table 4 indicates the calculated and experimental σ_0.2_, UTS, and elongation % values. Calculated σ_0.2_ values were close to the experimental ones. The ECAP samples calculated σ_0.2_ values deviated by 4.1–7.3% from the experimental one. This high accuracy of the Qiao et al. model [49] was also noted in the calculated σ_0.2_ of ECAP and ECAP combined with extrusion of Al-1080 and 1050 samples with a deviation range of 1.1–3.6% from the experimental results [16,49]. The accuracy of the Qiao et al. model [49] is considered higher than those used previously [10,47]. The ECAPed Al-1060 calculated yield strength [10] obtained based on the Hansen N model [57] has a deviation ranging from 4.4 to 27.8% from the experimental one. Moreover, the ECAPed Al-1070 calculated yield strength obtained based on the model previously reported [58,59,60] deviated by 5.1–11.2% from the experimental results [47]. The deviation range noted in Tolaminejad B et al.’s results [47] was higher than that of 2–8.4% obtained from the original model of Petryk H et al. [58] relative to the σ_0.2_ results of ECAPed Al99.99 [13]. Noting that the experimental results of σ_0.2_ did not indicate any saturation in σ_0.2_ [13], as assumed by the model [58].

Interestingly, the Petryk H et al. [58] results were based on the previous works [59,60] to calculate the strength of CECed and HPTed samples. However, the estimated yield strength of Al99.99 CECed samples depended on the same parameters used for the ECAP samples. Therefore, the deviation of calculated yield strength from the experimental one obtained from previous work [22] ranged from 23 to 31.7% after CEC under strain from 0.9–5. Therefore, Petryk H et al. [58] scaled the calculated yield strength values by 0.77 to reduce the deviation range from the experimental results [22] down to 0.29–4.1%.

The σ_0.2_ calculated values of the CEC samples in the present work indicate a deviation of 1.8–3.9% from the experimental values, which confirms the accuracy of using the Qiao et al. model [49]. Therefore, the Qiao et al. model [49] can be more accurate in calculating the tensile σ_0.2_ of the ECAP and CEC Al-1080 samples with high accuracy. The Qiao et al. model [49] accuracy is due to including the different microstructure parameters affecting the strength. Therefore, the Qiao et al. model [49] depends mainly on the microstructure parameters that can extracted accurately through the current work, not on a previous one that indicates a high deviation, as noted previously [10,47,58].

The dislocation density strengthened the ECAP and CEC samples by 63.9–47.7% and 64.7–52.4%. The contribution of the dislocation density decreases with further ECAP and CEC processing. The influence of the dislocation density in the strengthening of ECAPed and CECed samples was close in both cases after the first pass or cycle due to the close values of the dislocation density, as shown in Table 3. However, due to the smaller grain size and higher HAGBs in the ECAP samples, as shown in Figure 10 and Figure 11 and Table 2, their influence on the strength becomes more effective, so ECAP samples have higher strength than the CECed ones. The ECAP samples calculated tensile σ_0.2_ was higher by 2.2, 11.8, and 10% than that of the CEC samples processed under the same strain of 1.08, 5.4, and 10.8, respectively.

The experimental UTS and σ_0.2_ of the ECAP samples were higher by 11.5–20.6 and 2.6–16.4% than those of the CEC samples, confirming the calculating results. The higher values of the ECAP sample strength over those of the CEC one processed under the same strain were also noted from the comparison between previous works [8,9,21,22,61]. The compression yield strengths of the ECAPed Al-1050 samples processed using routes Bc and C under the strain of one, two, and eight [8,9] were higher by 18.2–14.1%, 14.6–9.4%, and 34.2–10.6% than those of the CEC Al-1050 samples processed under the same strain [21]. Moreover, the compression σ_0.2_ of the ECAPed high-purity Al samples [61] was higher by 51.8 and 49.2% than that of CEC high-purity Al samples deformed under strain of one and two [21]. The ECAP sample’s higher strength has also been noted in the case of the high-purity Al samples [22,61]. The compression σ_0.2_ of the ECAPed high purity Al samples processed under the strain of one to five [61] was higher by 58.2 and 44.4% than that of the CEC one deformed under the strains of one and five [22]. This observation is due to the smaller grain size and higher HAGBs in the ECAP samples. Therefore, the ECAP processed Al and Al alloys have higher strength than the CEC one, as noted from the current results and confirmed by the previous works.

The elongation of the ECAP and CEC samples decreased when increasing the number of passes and cycles. The elongation of the ECAP samples decreased by 44.8% after the first pass, then by 20.5% and 15.4% after 5 and 10 passes. Moreover, the elongation of the CEC samples decreased by 43%, 21.2, and 14.2% after 1, 5, and 10 cycles. The decrease in the tensile elongation also occurred in the case of ECAPed Al99.99, Al-1100, and Al-1080 [12,13,14,15,16,29]. However, the elongation behavior sometimes deviated from the continuous decrease with the number of passes [12,13,14,15]. The ECAPed sample’s elongation decreased sharply after the first pass, then slightly increased and decreased, with a final decrease with further processing [12,13,14,15]. This behavior is due to the stable equilibrium in the microstructure and the grain growth [12,13,14,15].

Although many previous works were available about the behavior of the ECAPed Al elongation, there is a shortage of data on the tensile elongation of the CEC-processed Al samples. However, the elongation of the CEC AA5052 samples has the same behavior noted in the current study. The CECed AA5052 elongation was decreased by 38.6%, then by 2.8%, and, finally, by 45.3% after processing under strain of 0.9, 1.8, and 2.7, respectively [28]. Interestingly, the elongation of CEC samples was slightly higher, by 3.4–3.9%, than that of the ECAP. The ECAPed and CECed sample’s elongation decrease is due to the influence of the strain hardening [28,62]. Moreover, the resistance of the fine grain and grain boundaries to dislocation motion also contributes to decreases in the elongation. Therefore, the smaller grain size with a higher grain boundary misorientation angle of the ECAP samples explains the relatively slightly higher elongation of the CEC samples.

The elongation change can also be related to the values of average boundaries with misorientation greater than or equal to 15° (%) and dislocation density, shown in Table 2 and Table 3. After one pass and cycle, the dislocation density of the ECAP and CEC were so close to each other, but the value of the average boundaries with misorientation greater than or equal to 15° (%) of the ECAP sample was 40%, which is higher than that of the 38.5% in the case of the CEC sample for one cycle. Moreover, the decrease in the grain size to 19.8 and 23 µm after ECAP and CEC after one pass and cycle increases the resistance of the dislocation motion in the ECAP and CEC samples. However, due to the smaller grain size and higher percentage of HAGBs in the ECAP sample, it has a relatively lower elongation than the CEC one. The average boundaries with misorientation greater than or equal to 15° (%) values increase to 83.2 and 77% with average grain sizes of 0.3 and 0.38 µm after ten passes and cycles of the ECAP and CEC process. Therefore, an obvious hindrance for the dislocation motion occurs, which decreases the elongation to 15.7 and 16.3%. However, due to the smaller grain size and higher percentage of the HAGBs of the ECAP sample after 10 passes, it has a relatively lower elongation than the CEC sample. Therefore, the decrease in the elongation can be related to the resistance of dislocation motion that increases with further grain refinement and the increase in HAGBs.

## 4. Conclusions

The present study revealed the following conclusions.

1. The ECAP processing of Al-1080 bars was much easier to perform and repeat up to multiple passes than the CEC processing under the same strain.
2. ECAP load–displacement behavior was smoother than that of CEC, characterized by complex steps. The peak load of the CEC was higher, by 218.8–265.4%, than that of the ECAP processing under the same strain, which indicates the effectiveness of ECAP processing using a lower load.
3. The ECAP sample’s microstructure has a high degree of homogeneity under a strain of 10.8 relative to the shear band structure of the CEC samples.
4. The ECAP and CEC processing of Al-1080 samples could produce UFG microstructure samples. However, the ECAP samples have smaller grain sizes with higher percentages of HAGBs.
5. UTS and σ_0.2_ of the ECAP Al-1080 samples were higher by 11.5–20.6 and 2.6–16.4% due to the smaller grain size by 13.9–44%, with HAGBs higher, by 3.8–8.1%, than the CECed samples. However, the CEC samples have a slightly higher elongation, by 3.4–3.9%, than those of the ECAP.
6. The σ_0.2_ values of the ECAP and CEC samples were accurately calculated based on a mathematical model with a deviation range of 1.8–7.3% from the experimental one.

## Figures and Tables

**Table 1:** Chemical composition of the commercially pure aluminum Al-1080 (wt.%).

Chemical Composition (wt.%)										
ISO	DIN	Si	Fe	Cu	Mn	Mg	Zn	Ti	Others	Al
1080A	Al 99.8	<0.15	<0.15	<0.03	<0.02	<0.02	<0.06	<0.02	<0.02	>98.8

**Table 2:** EBSD microstructure parameters of the different samples.

Sample	Average Grain Size (µm)	Grain Size Range (µm)	Average Misorientation Angle (°)	Average Boundaries with Misorientation Greater than or Equal to 15° (%)
A1-1080	414	-	-	-
ECAP 1 pass	19.8	1.39–22.66	21.9	40
ECAP 5 passes	1	0.42–4.49	32	75.8
ECAP 10 passes	0.3	0.15–0.84	35.2	83.2
CEC 1 cycle	23	1.8–27.9	19.6	38.5
CEC 5 cycles	1.8	0.53–4.86	31	73.3
CEC 10 cycles	0.38	0.21–0.96	33.5	77

**Table 4:** Summary of the experimental and calculated tensile properties of the ECAP and CEC Al-1080 samples.

Sample	Experiential σ_0.2_ (MPa)	Calculated σ_0.2_ (MPa)	UTS (MP)	Elongation %
**A1-1080**	31.5	-	47.6	42.3
**ECAP 1 pass**	104.6	100	113.9	23.3
**ECAP 5 passes**	133.2	126	138.5	18.6
**ECAP 10 passes**	175.9	163	183.3	15.7
**CEC 1 cycle**	101.9	98.1	105.5	24.1
**CEC 5 cycles**	117.1	113	121.9	19
**CEC 10 cycles**	151.2	148	155.9	16.3

## Data Availability

The data presented in this study are available on request from the corresponding author. The data are not publicly available due to their extremely large size.
